# Supplementary material for: Dual-mechanism based CTLs infiltration enhancement initiated by Nano-sapper potentiates immunotherapy against immune-excluded tumors
Source: Nat Commun. 2020 Jan 30;11:622. doi: 10.1038/s41467-020-14425-7 (PMC6992734; doi:10.1038/s41467-020-14425-7)
Supplement: Supplementary file 1 — Supplementary Information [file 41467_2020_14425_MOESM1_ESM.pdf]

Supplementary Information for

Dual-Mechanism Based CTLs Infiltration Enhancement Initiated by Nano-sapper Potentiates Immunotherapy against Immune-Excluded Tumors

Huang et al.

a

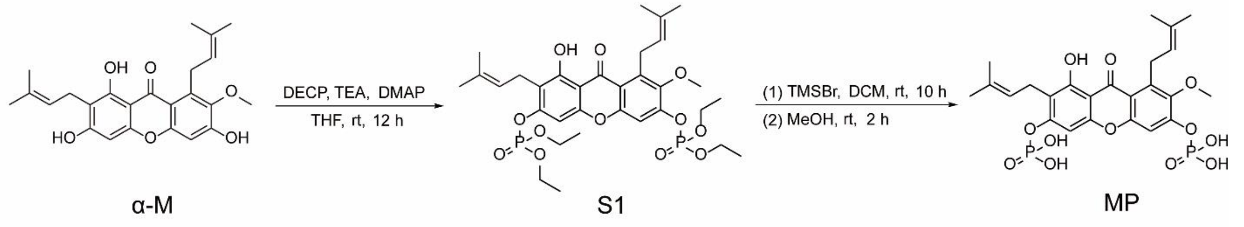

b

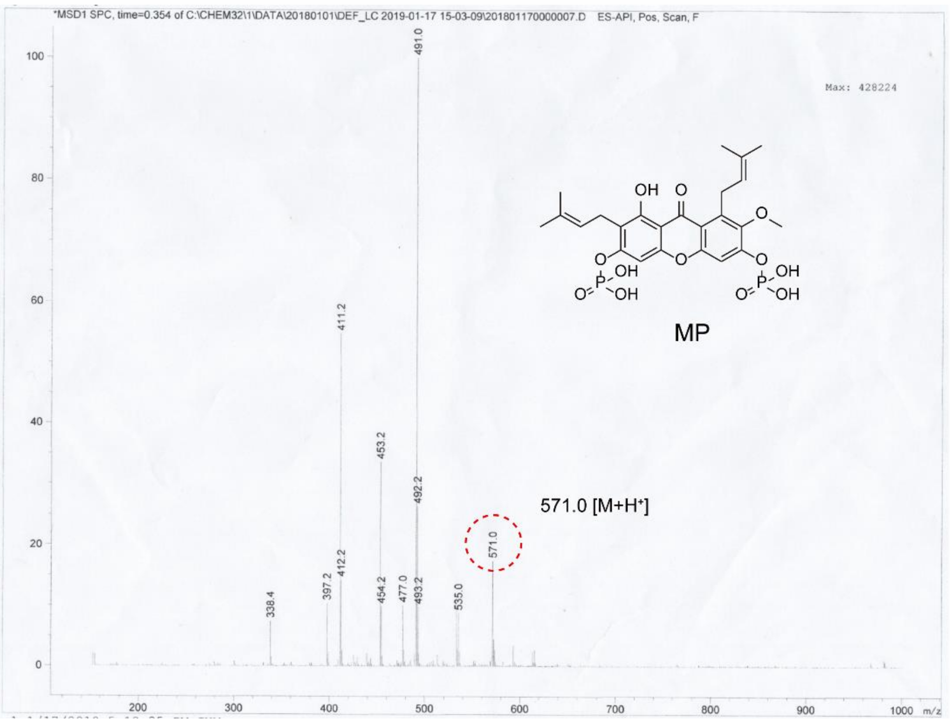

Supplementary Figure 1 The synthesis of MP. **a** The synthetic route of MP; **b** The mass spectra of MP. MP, α-mangostin phosphate

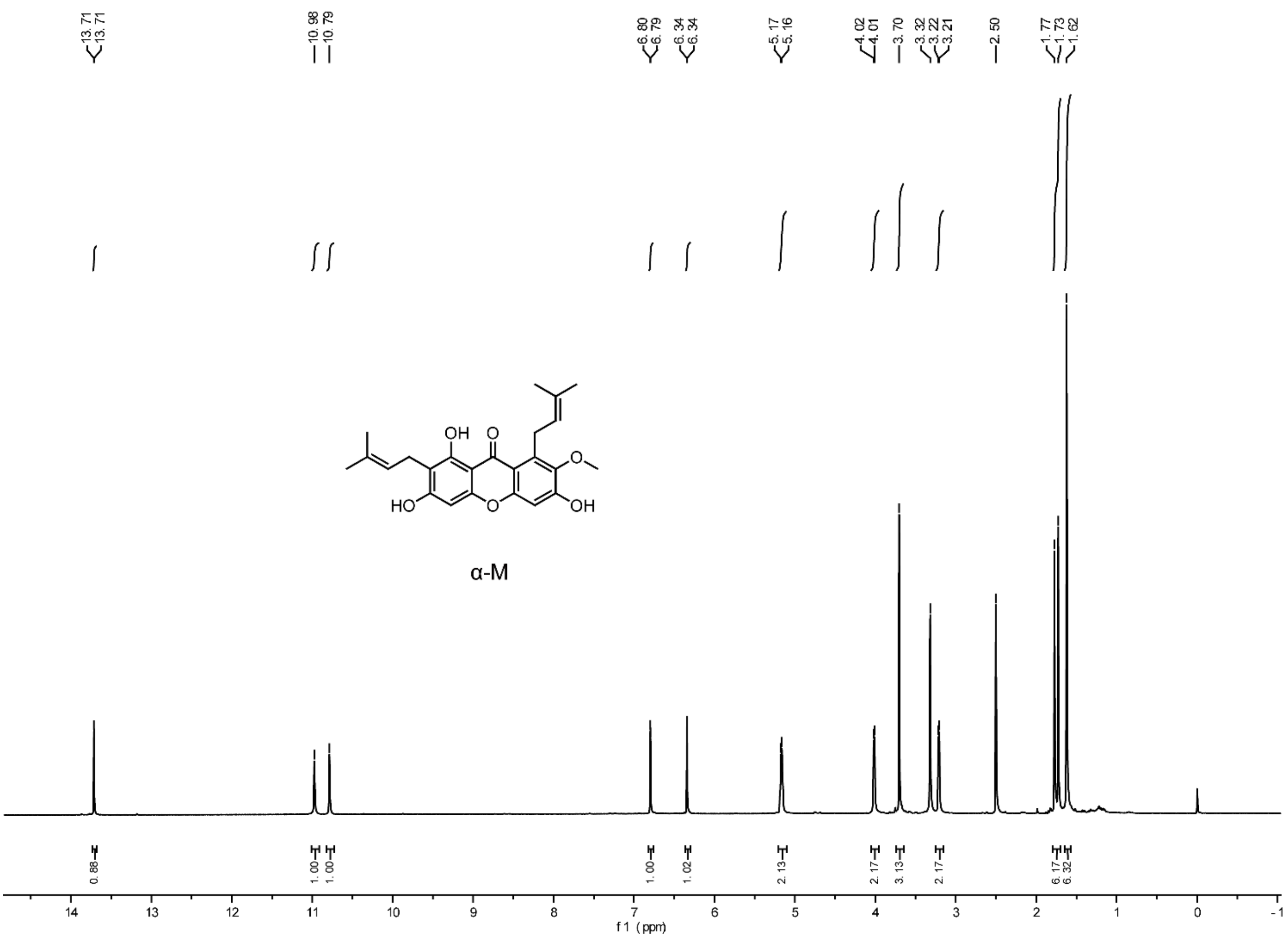

Supplementary Figure 2 ^{1}H-NMR spectra of α-M. ^{1}H NMR (600 MHz, DMSO-d_6): δ = 13.71 (d, J = 2.3 Hz, 1H), 10.98 (s, 1H), 10.79 (s, 1H), 6.80 (d, J = 2.3 Hz, 1H), 6.34 (d, J = 1.8 Hz, 1H), 5.17 (d, J = 7.1 Hz, 2H), 4.02 (d, J = 5.2 Hz, 2H), 3.70 (s, 3H), 3.21 (d, J = 6.7 Hz, 2H), 1.75 (d, J = 27.8 Hz, 6H), 1.62 (s, 6H).

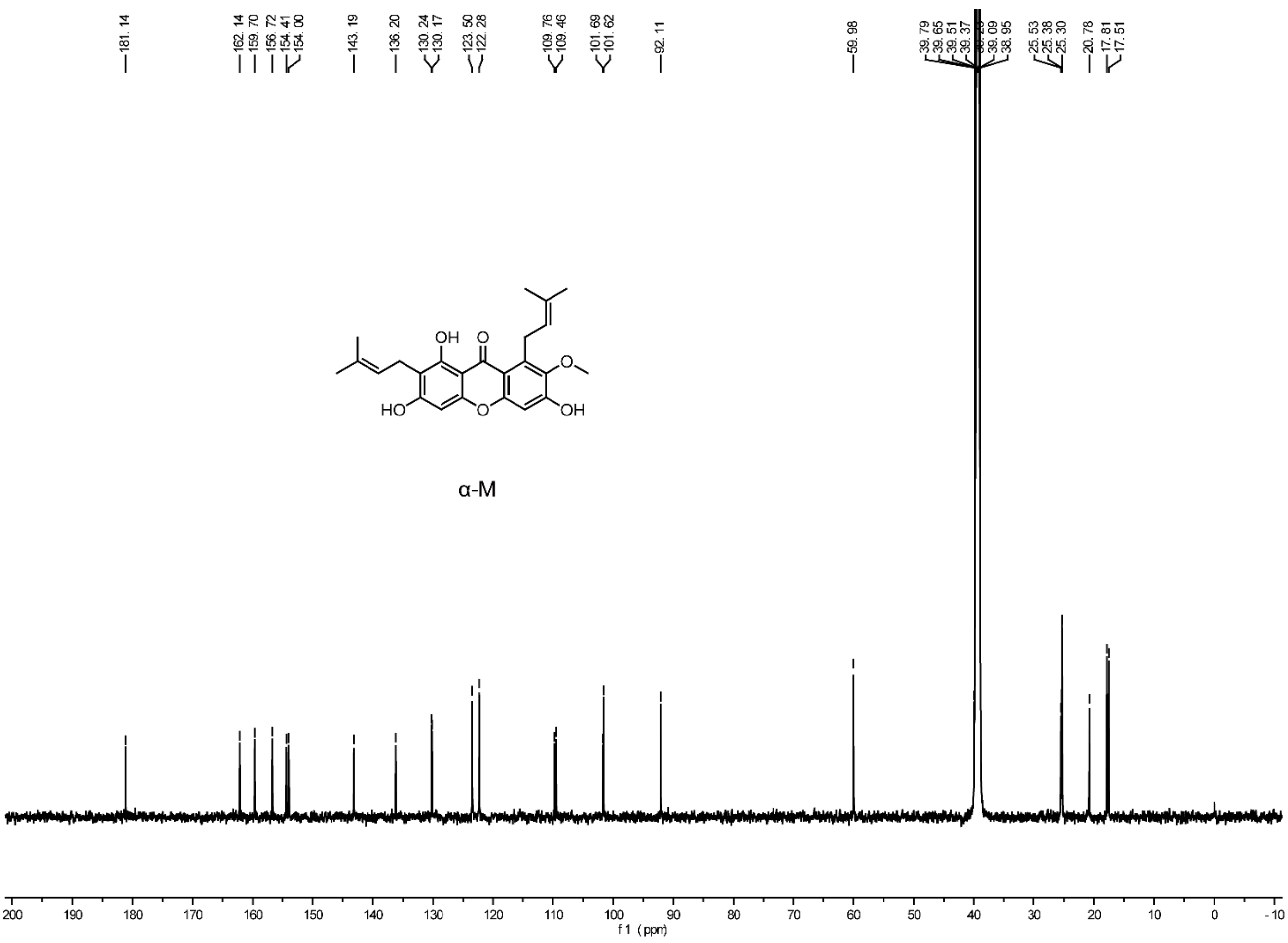

Supplementary Figure 3 ^{13}C-NMR spectra of α-M. ^{13}C NMR (151 MHz, DMSO-d_6): δ = 181.14, 162.14, 159.70, 156.72, 154.41, 154.00, 143.19, 136.20, 130.24, 130.17, 123.50, 122.28, 109.76, 109.46, 101.69, 101.62, 92.11, 59.98, 25.53, 25.38, 25.30, 20.78, 17.81, 17.51.

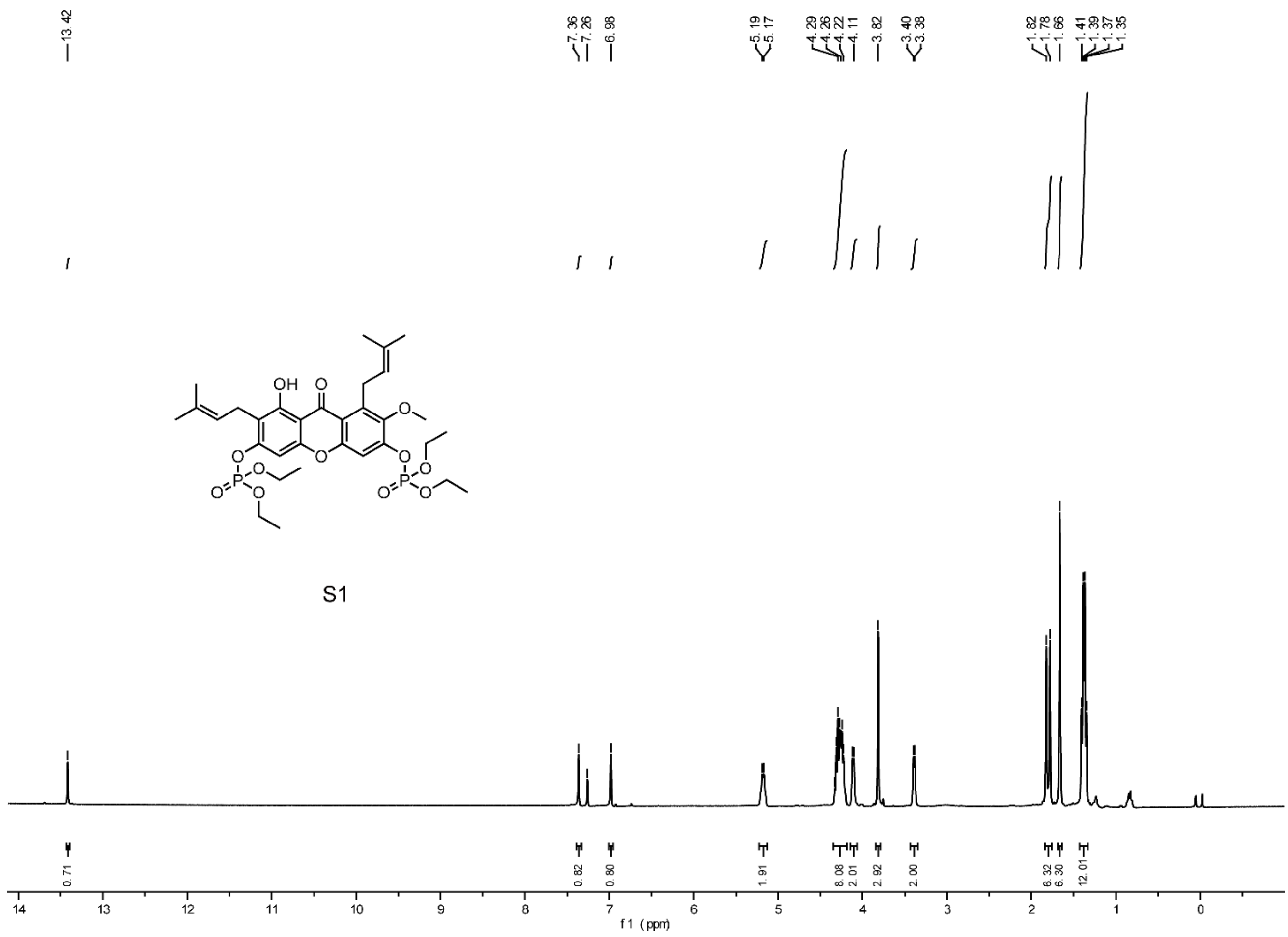

Supplementary Figure 4 ^{1}H-NMR spectra of S1. ^{1}H NMR (600 MHz, $CDCl_3$): δ = 13.43 (s, 1H), 7.37 (s, 1H), 6.99 (s, 1H), 5.19 (d, J = 7.4 Hz, 2H), 4.36 – 4.20 (m, 8H), 4.12 (d, J = 5.8 Hz, 2H), 3.83 (s, 3H), 3.40 (d, J = 6.4 Hz, 2H), 1.82 (s, 3H), 1.78 (s, 3H) 1.67 (s, 6H), 1.39 (dd, J = 15.9, 7.7 Hz, 12H).

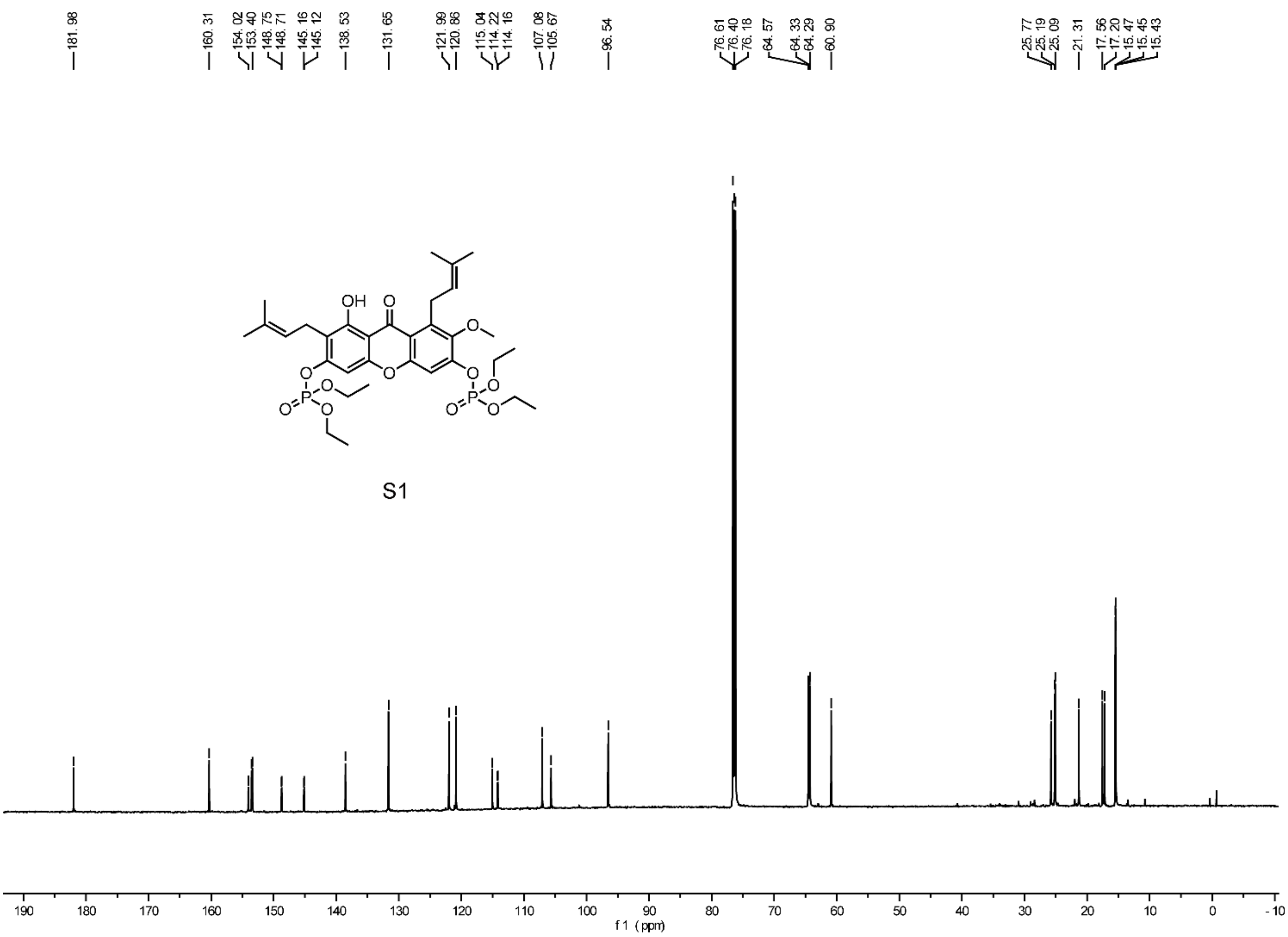

Supplementary Figure 5 ^{13}C-NMR spectra of S1. ^{13}C NMR (151 MHz, $CDCl_3$): δ = 181.98, 160.31, 154.05, 154.02, 153.53, 153.40, 148.75, 148.71, 145.16, 145.12, 138.53, 131.65, 121.99, 120.86, 115.04, 114.22, 114.16, 107.08, 105.67, 96.54, 76.61, 76.40, 76.18, 64.57, 64.53, 64.33, 64.29, 60.90, 25.77, 25.19, 25.09, 21.31, 17.56, 17.20, 15.47, 15.45, 15.43.

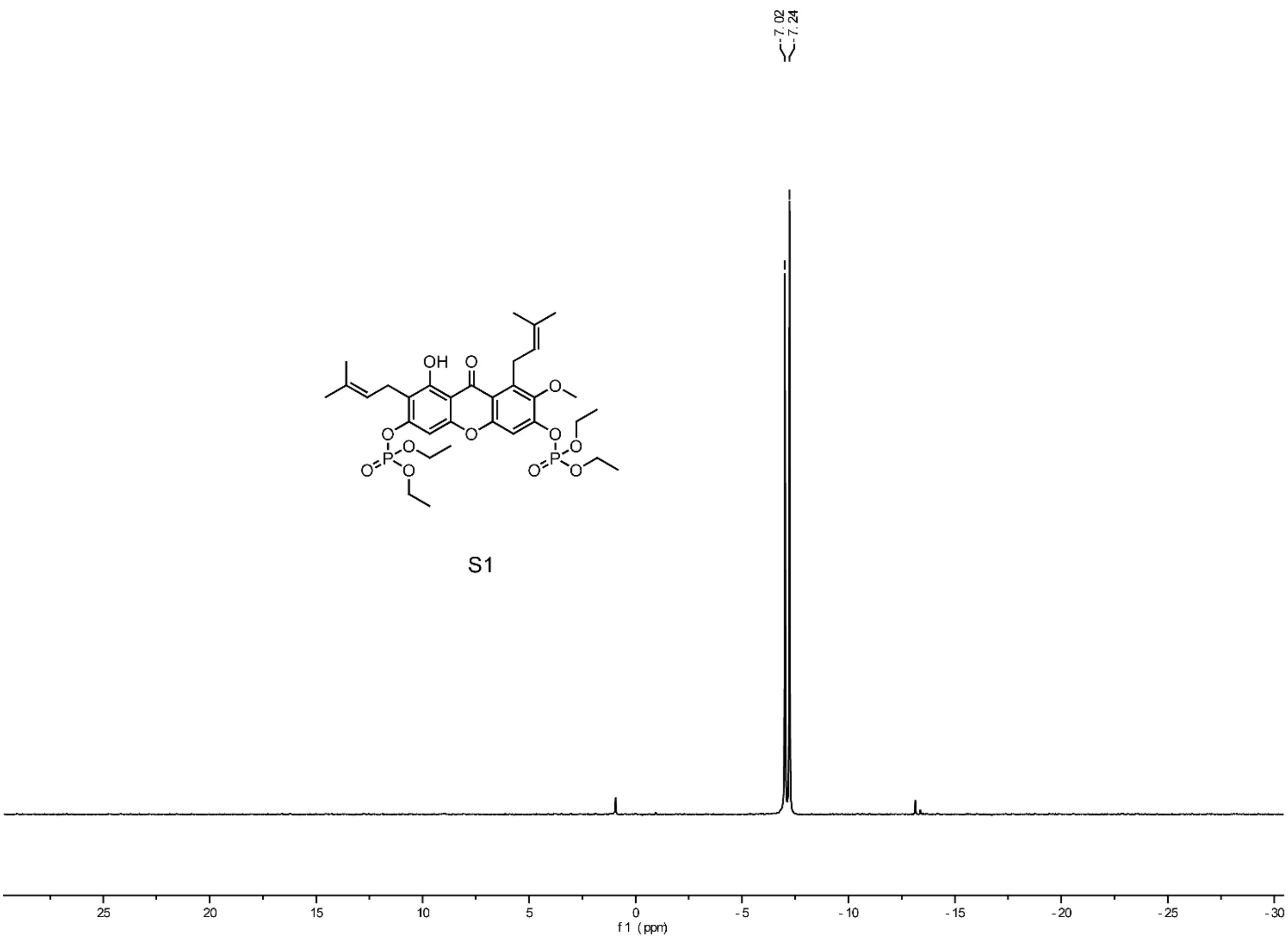

Supplementary Figure 6 ^{31}P-NMR spectra of S1. ^{31}P NMR (162 MHz, $CDCl_3$): δ = -7.02, -7.24 ppm.

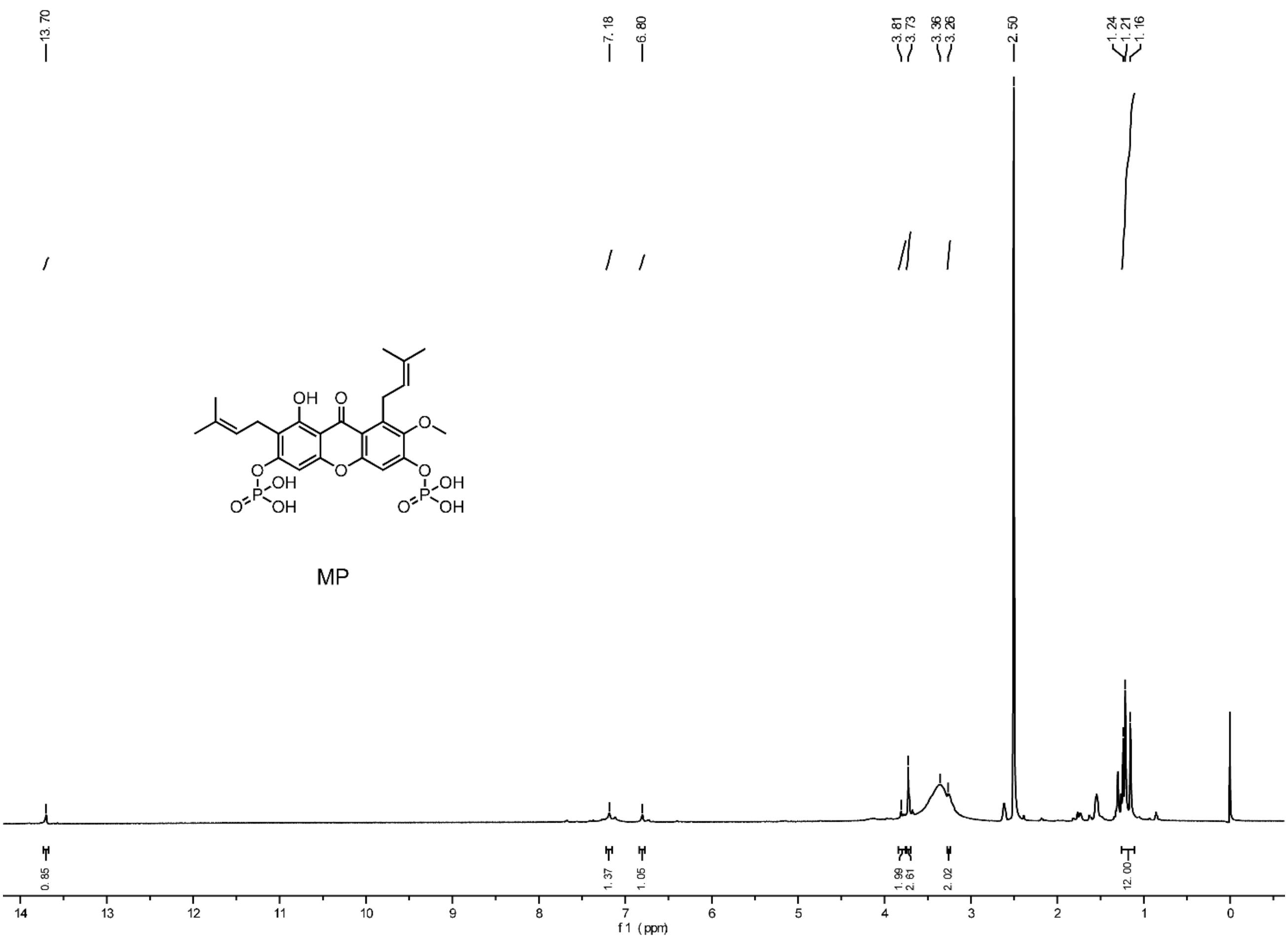

Supplementary Figure 7 ^{1}H-NMR spectra of MP. ^{1}H NMR (600 MHz, DMSO-d_6): δ = 14.46 (s, 1H), 7.92 (s, 1H), 7.55 (s, 1H), 4.45 (br s, -POH), 2.15 – 1.70 (m, 12H);

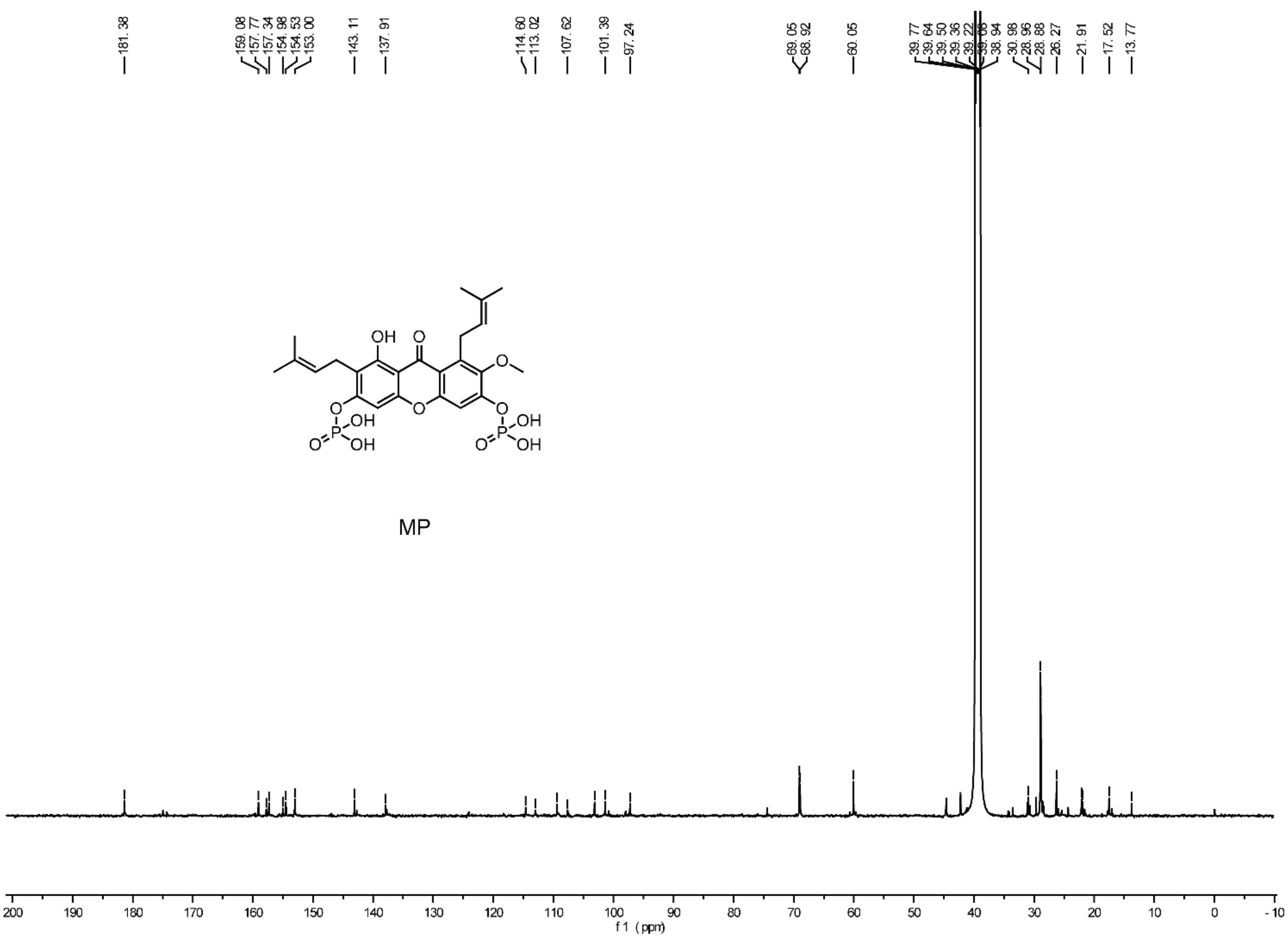

Supplementary Figure 8 ^{13}C-NMR spectra of MP. ^{13}C NMR (150 MHz, $CDCl_3$): δ = 150.3, 147.6, 133.6, 133.1, 132.5, 132.1, 131.9, 129.4, 127.3, 127.2, 127.1, 126.3, 125.6, 125.2, 124.7, 117.5, 117.2, 112.3, 98.0, 65.3, 14.5 ppm.

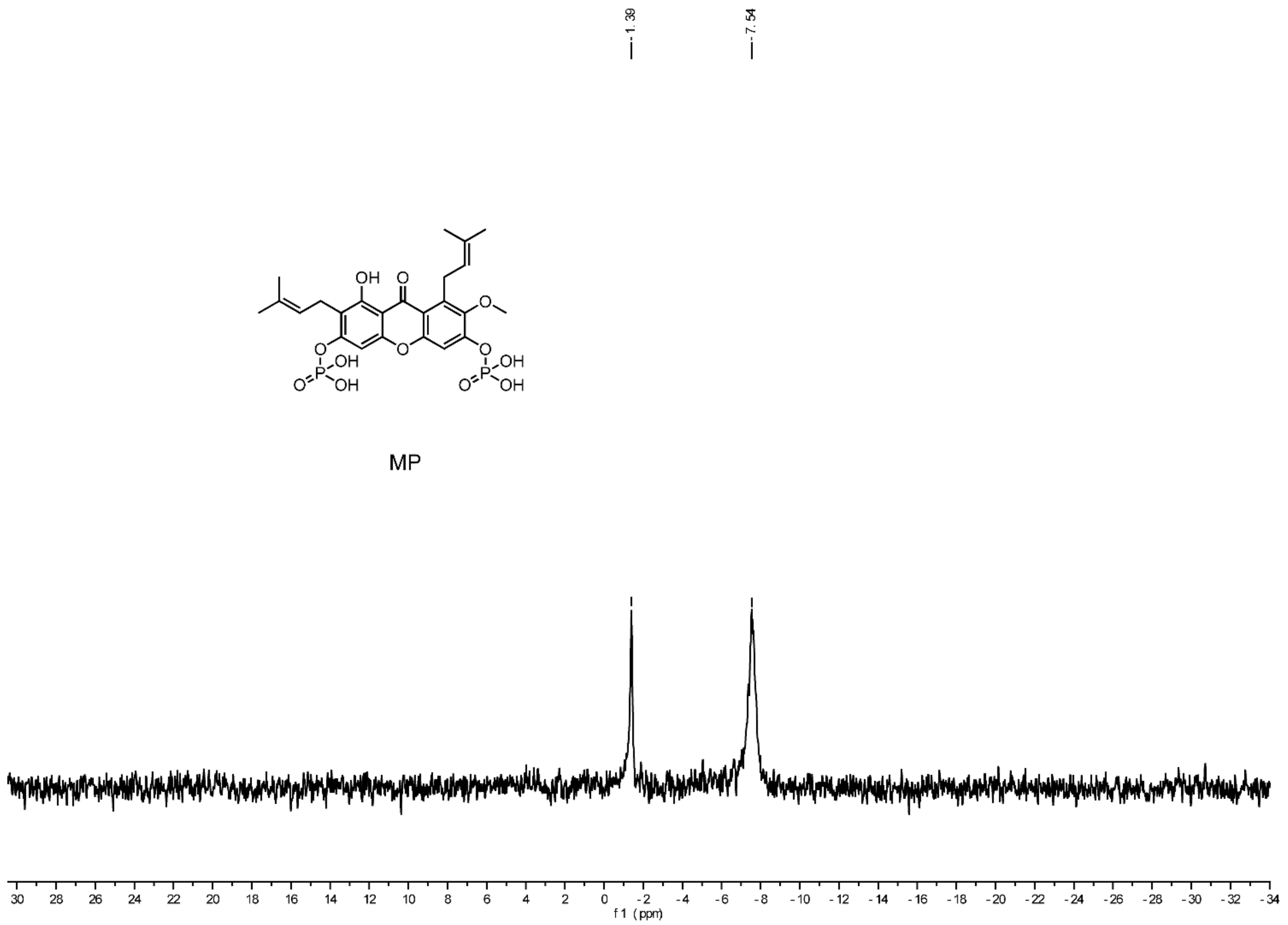

Supplementary Figure 9 ^{31}P-NMR spectra of MP. ^{31}P NMR (162 MHz, DMSO-*d6*) δ = -1.39, -7.54 ppm.

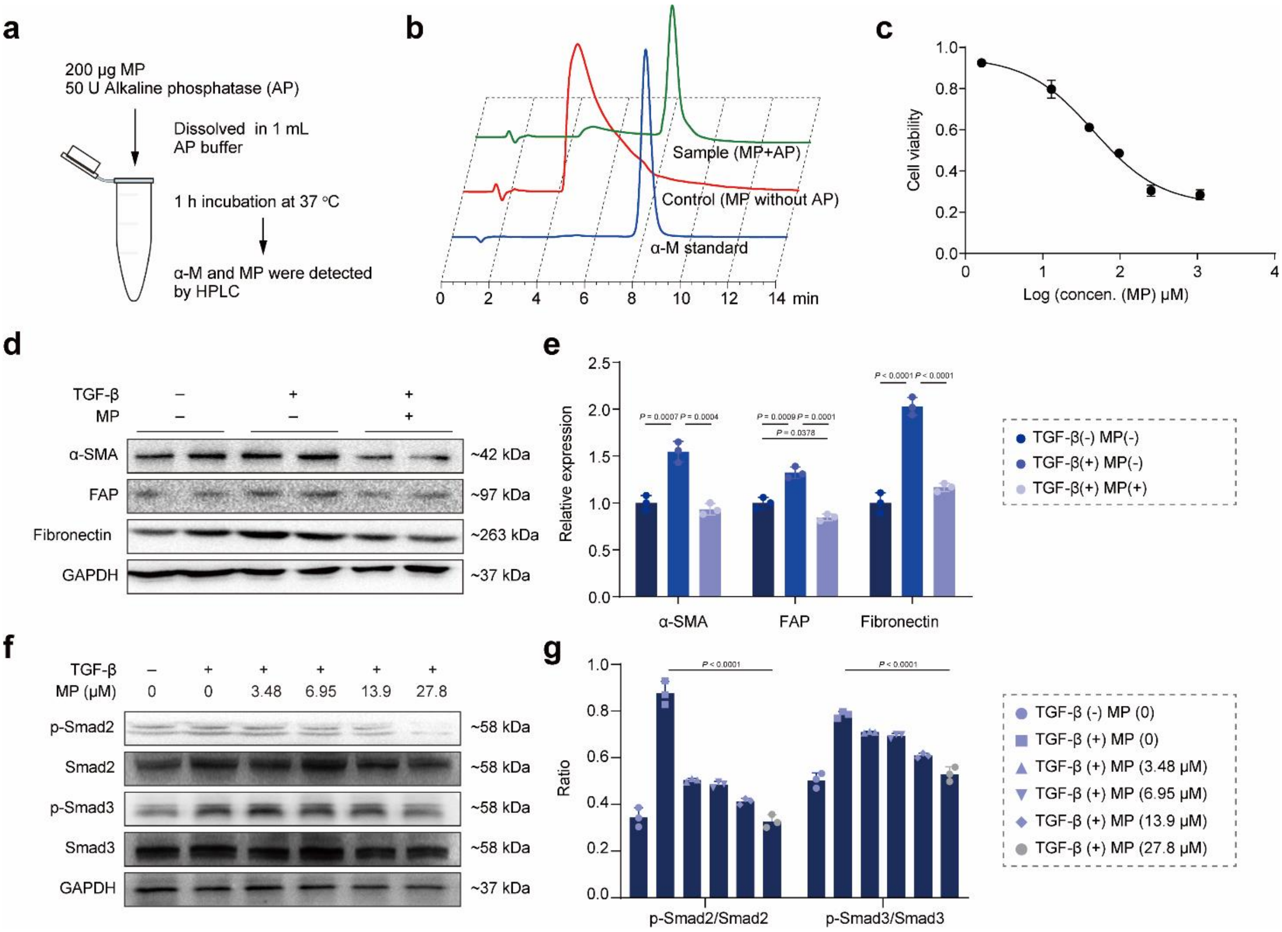

Supplementary Figure 10 The conversion of MP to α-M and the reversion of CAFs without inducing apparent cytotoxicity by MP. **a,b** General procedures and HPLC spectrums of the solution containing free MP and AP after 1 h incubation at 37 °C. **c** The cytotoxicity of MP in activated NIH3T3 cells (pre-treated with TGF-β at 10 ng/mL for 24 h). (*n* = 3 independent experiments). **d,e** The expression of α-SMA, FAP and fibronectin in activated NIH3T3 after incubation with MP at 27.8 μM for 24 h and semi-quantitatively analyzed by ImageJ. (*n* = 3 independent experiments). **f,g** Phosphorylation of Smad2 and Smad3 in activated NIH3T3 were inhibited after incubation with MP for 24 h. (*n* = 3 independent experiments). Data are presented as mean ± s.d., One-way ANOVA with Bonferroni multiple comparisons post-test was used for **e,g**. Error bars represent s.d.. Source data of Supplementary Fig. 10d and f are provided as a Source Data file.

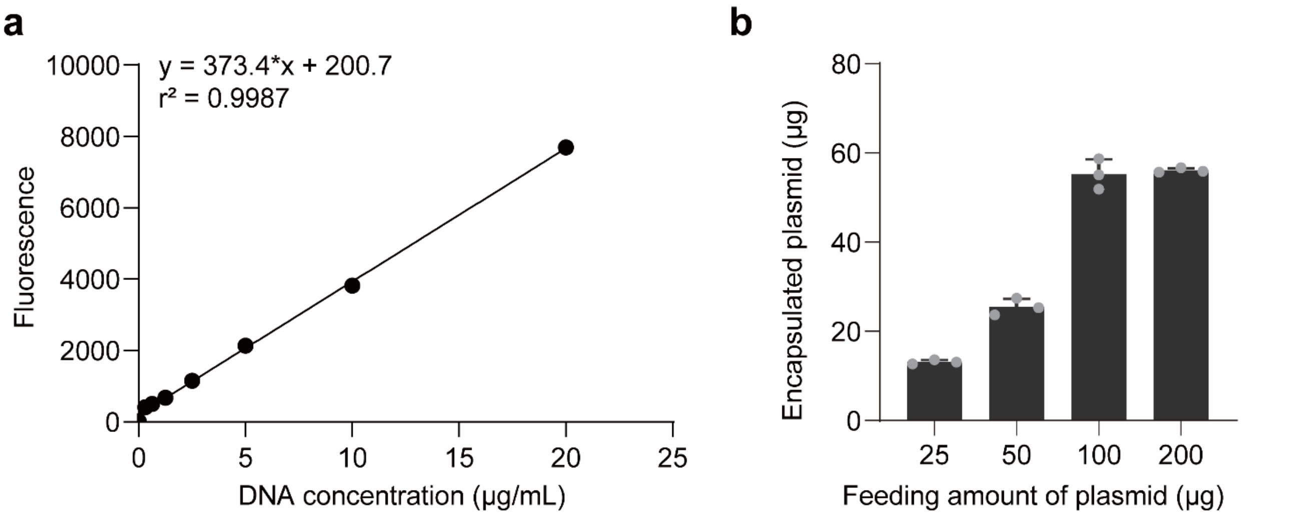

Supplementary Figure 11 The determination of plasmid concentration. **a** Concentration curve from 0.0 to 20 μg/ml with linear regression analysis. **b** The encapsulated plasmid detected by Hoechst 33258 staining. *n* = 3 independent samples Data are presented as mean ± s.d. Error bars represent s.d..

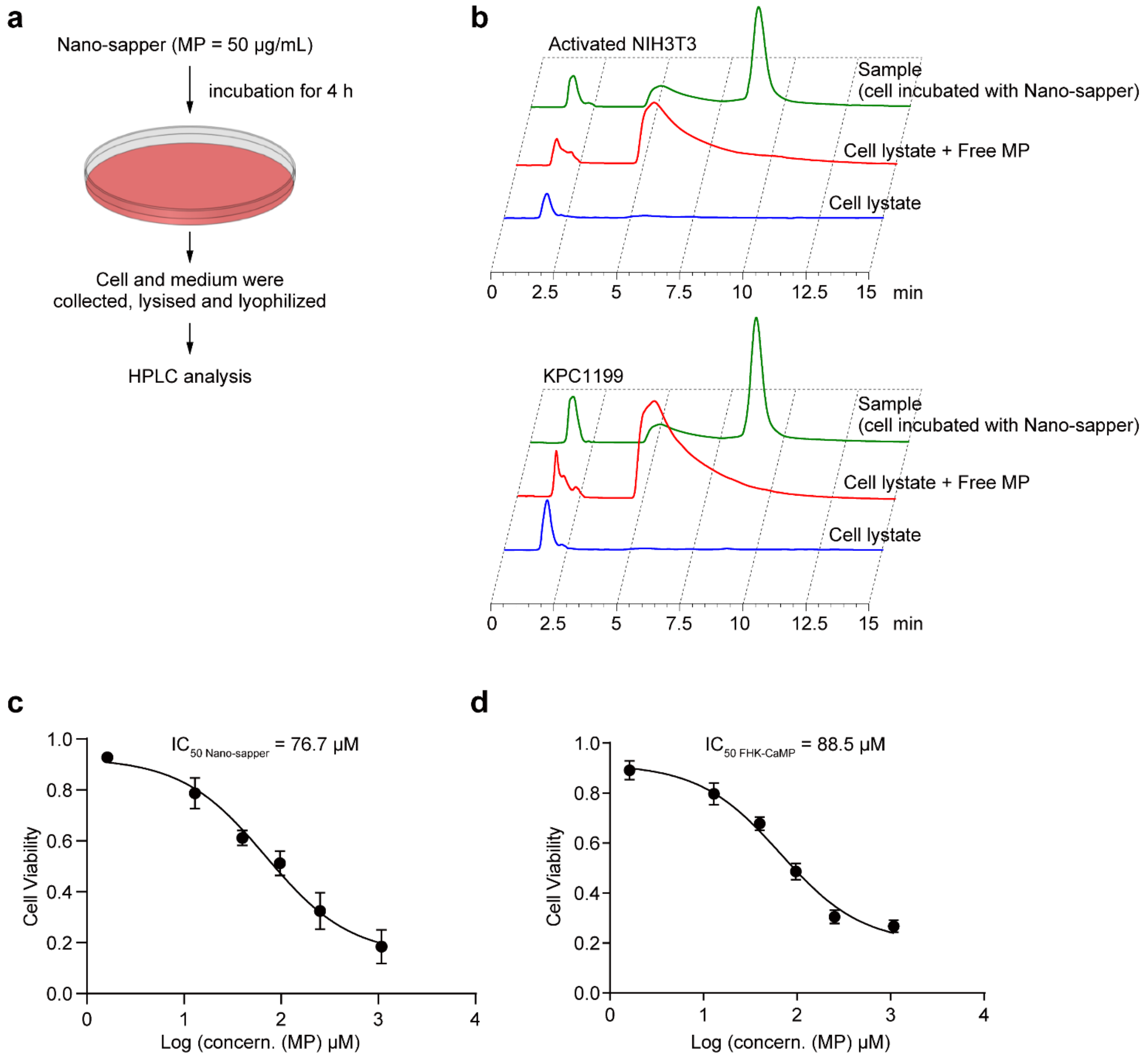

Supplementary Figure 12 The degradation and cytotoxicity of Nano-sapper. **a,b** General procedures and HPLC analysis of Nano-sapper incubated with activated NIH3T3 or KPC1199 cells. **c,d** Cytotoxicity of indicated nanoparticles with varied MP concentration were incubated with activated NIH3T3 for 24 h. Data are presented as mean ± s.d. (n = 3 independent experiments). Error bars represent s.d..

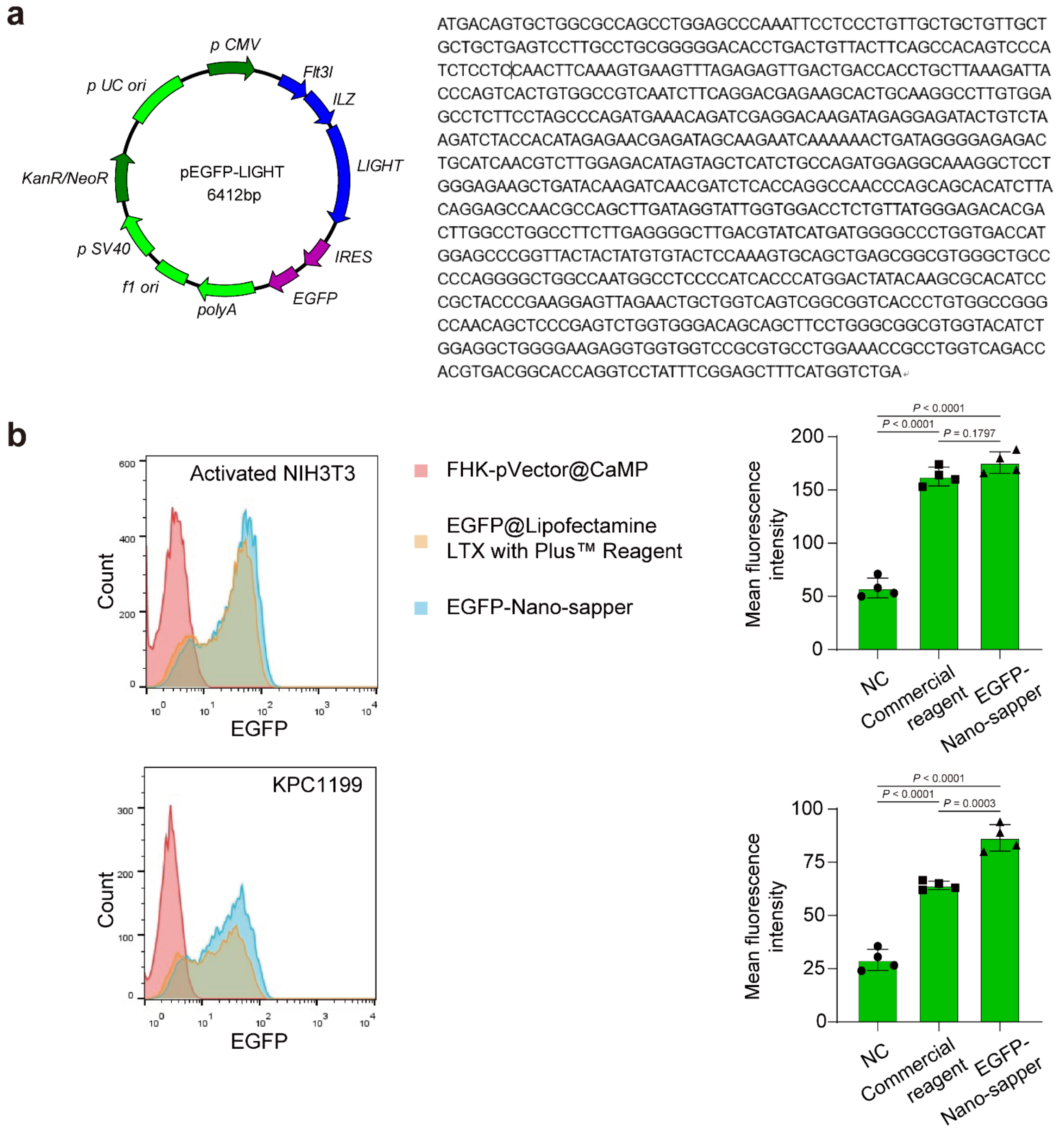

Supplementary Figure 13 Representative scheme of plasmid and EGFP expression with different treatments. **a** The sequence of plasmid simutaneously encoding LIGHT and EGFP. **b** The transfection efficiency determined by flow cytometry. Negative control (NC), the non-EGFP coding vector plasmid loaded nanoparticle (FHK-pVector@CaMP). Data are presented as mean ± s.d. (n = 3 independent experiments). One-way ANOVA with Bonferroni multiple comparisons post-test was used for **b**. Error bars represent s.d..

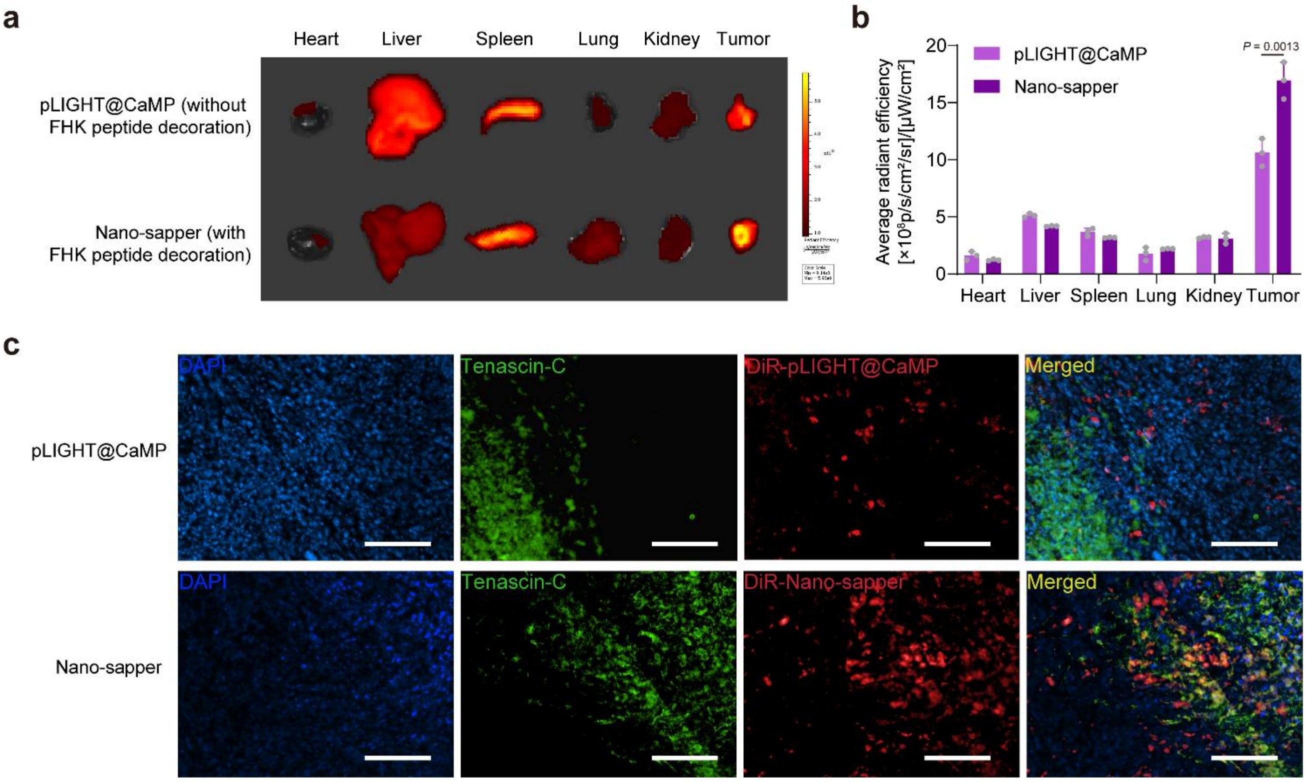

Supplementary Figure 14 The PDAC targeting ability of FHK peptide. **a** Major organs and tumors were harvested and imaged 24 h after DiR-labelled nanoparticles *i.v.* administrated. **b** Semi-quantitative analysis according to fluorescence signal intensity (n = 3 mice). **c** Tumors slices were stained with anti-Tenascin C antibodies. Paired Student's t test was used for **b**. Scale bars, 200 μm. Error bars represent s.d.. Source data of Supplementary Fig. 14b are provided as a Source Data file.

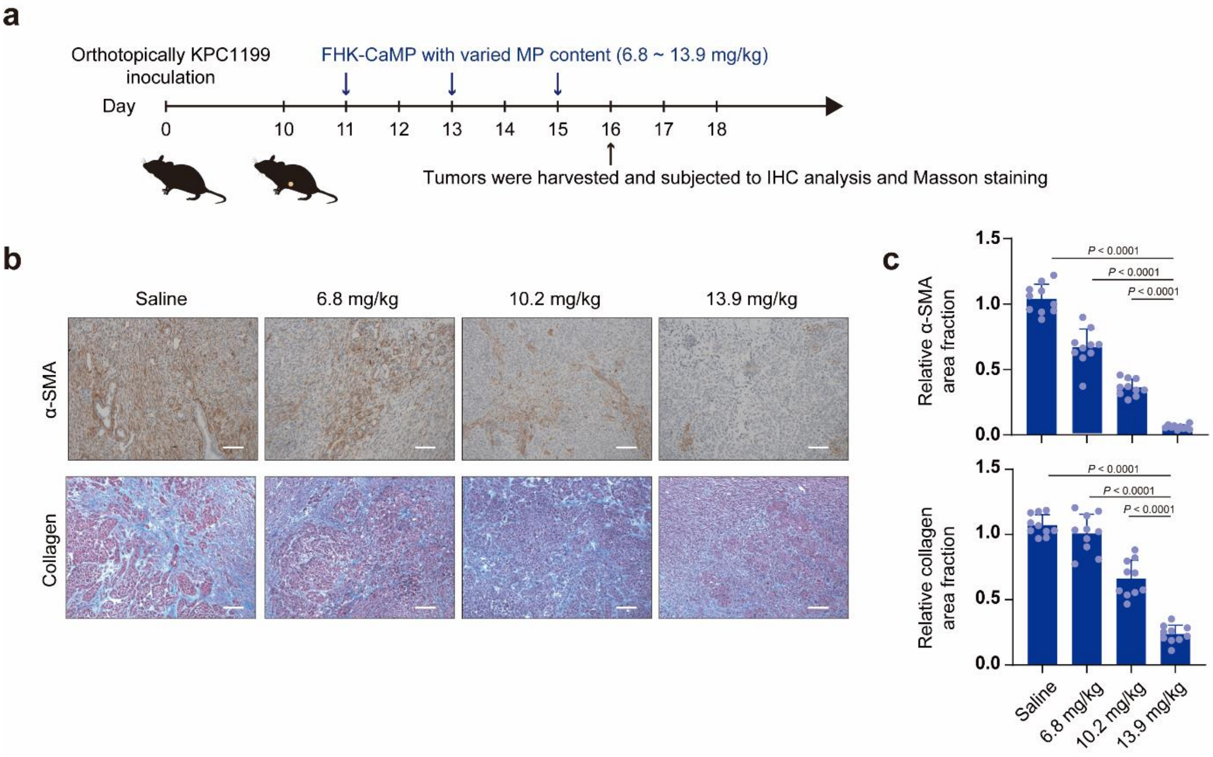

Supplementary Figure 15 The determination of the optimized dosage of FHK-CaMP**. a** Six-week-old male C57BL/6 mice were orthotopically inoculated with KPC1199 cells (1×10^6) on day 0. Saline and FHK-CaMP with different MP dosage (6.8, 10.2 or 13.9 mg/kg) were *i.v.* administrated on day 11, 13 and 15. On day 16, tumors were harvested and subjected to IHC analysis and Masson staining. **b,c** Tumor slices were stained for α-SMA and collagen, and determined by ImageJ. Scale bars, 100 μm. ($n = 10$ section images from three mice). Data are presented as mean ± s.d. One-way ANOVA with Bonferroni multiple comparisons post-test was used for **c**. Error bars represent s.d..

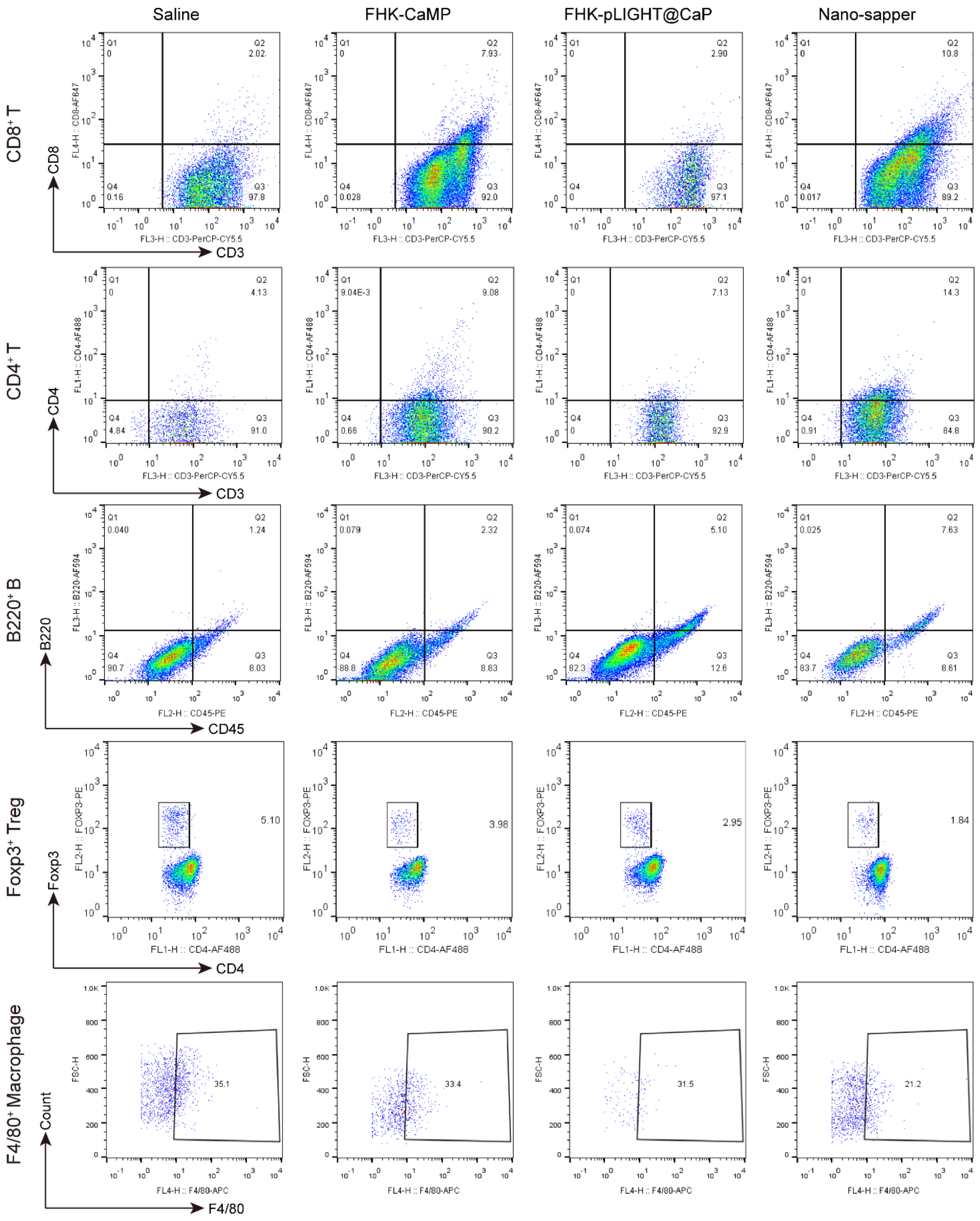

Supplementary Figure 16 The representative flow cytometry plots of CD45$^+$CD3$^+$CD8$^+$ T cells, CD45$^+$ CD3$^+$CD4$^+$ T cells, CD45$^+$B220$^+$ B cells in tumors while decrease the ratio of CD4$^+$Foxp3$^+$ Tregs and F4/80$^+$ macrophages.

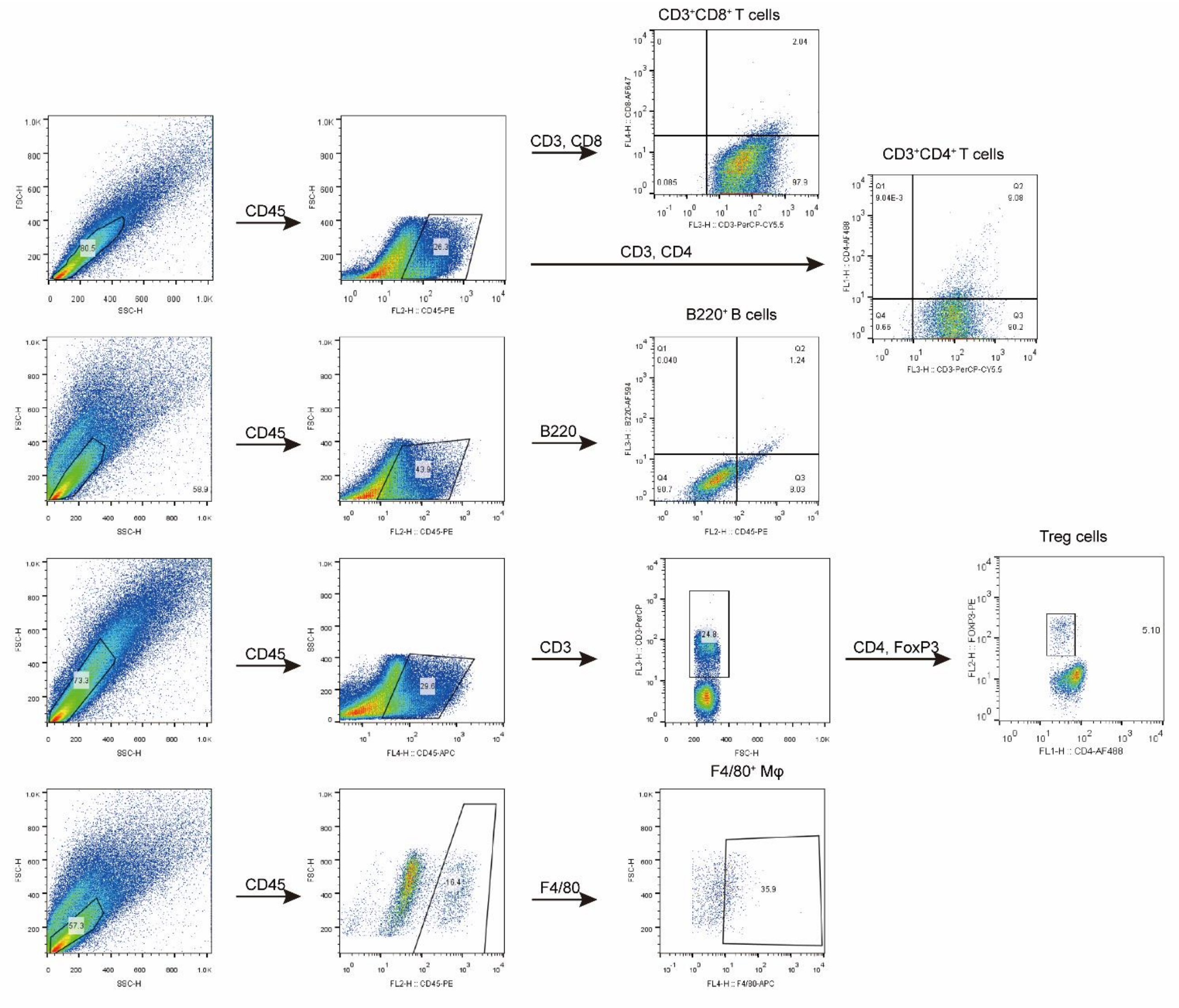

Supplementary Figure 17 Gating strategies used for flow cytometry analysis of immune cell population.

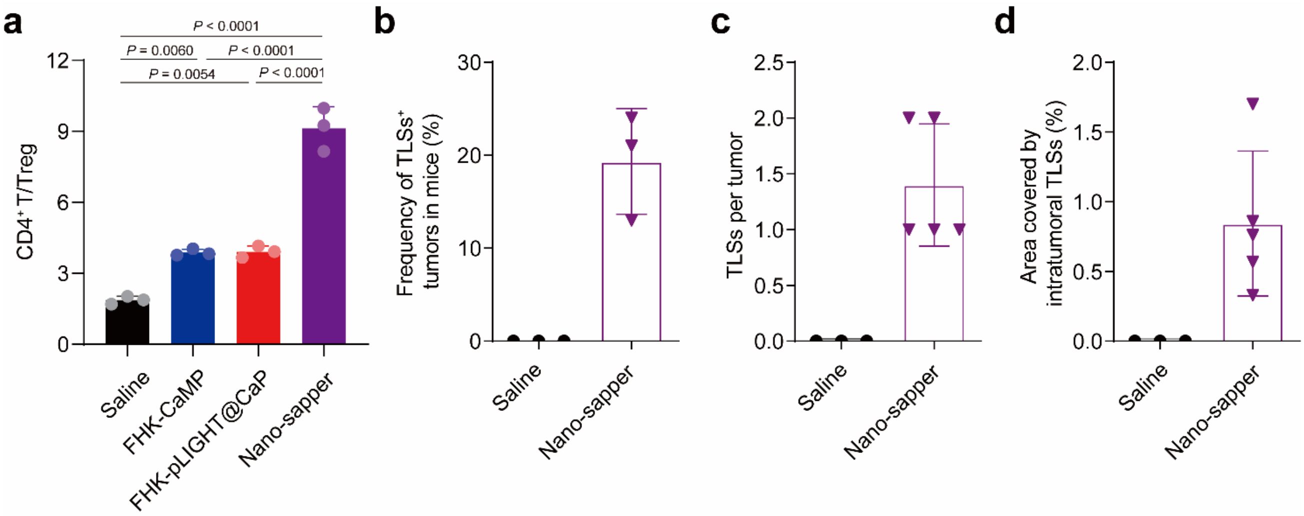

Supplementary Figure 18 Nano-sapper modulate ratio of lymphocytes within TME and the quantitative determination of TLSs. **a** Nano-sapper increase the $CD4^+$ T/Treg ratio in TME (n = 3 mice). **b** Frequency of $TLSs^+$ tumors in mice (n = 3 mice). **c** TLSs per tumor (n = 5 section images from three mice). **d** Area covered by intratumoral TLSs (n = 5 section images from three mice). Data are presented as mean ± s.d. One-way ANOVA with Bonferroni multiple comparisons post-test was used for **a**. Error bars represent s.d..

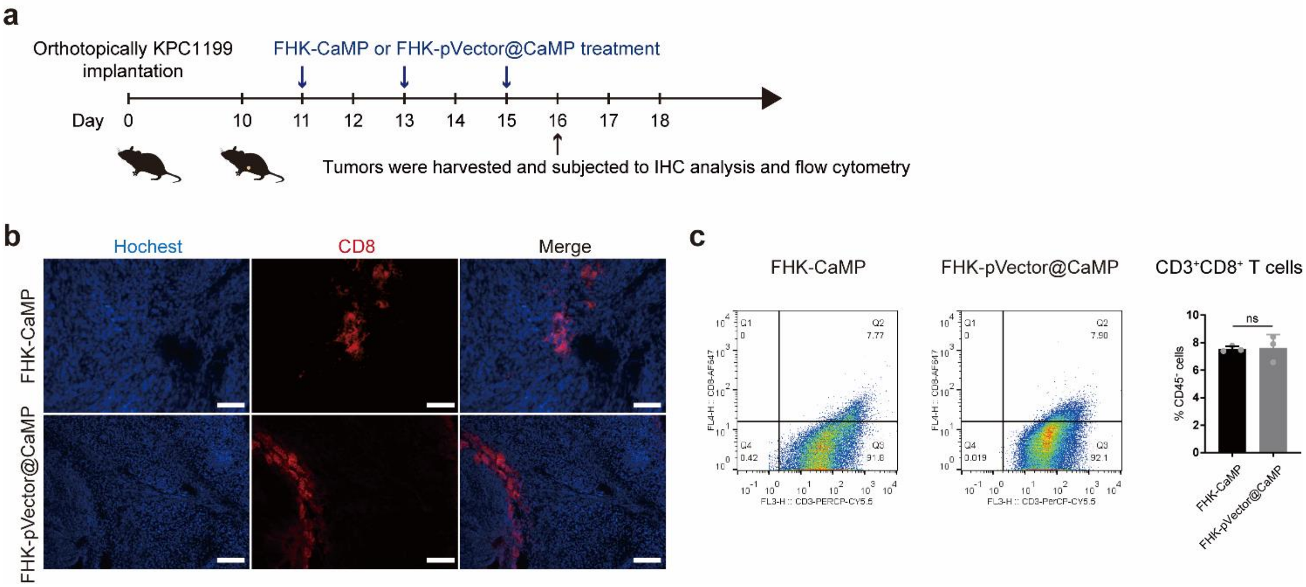

Supplementary Figure 19 The loaded plasmid did not induce observable variation in the infiltration of CD8$^+$ T cell. **a** Six-week-old male C57BL/6 mice were orthotopically inoculated with KPC1199 cells (1×10^6) on day 0. FHK-CaMP (MP = 13.9 mg/kg), FHK-pVector@CaMP (MP = 13.9 mg/kg, 25 μg plasmid per mouse) were *i.v.* administrated every other day (n = 3 mice). At day 16, tumors were extracted and subjected to IHC and flow cytometry assays. **b** Infiltration of CD8$^+$ T characterized by IHC. FHK-pVector@CaMP, nanoparticle loaded with scrambled LIGHT sequences-encoded vector plasmid. Scale bars, 100 μm. **c** Infiltration of CD8$^+$ T characterized by flow cytometry (n = 3 mice). Data are presented as mean ± s.d. One-way ANOVA with Bonferroni multiple comparisons post-test was used for **c**. ns, not significant. Error bars represent s.d..

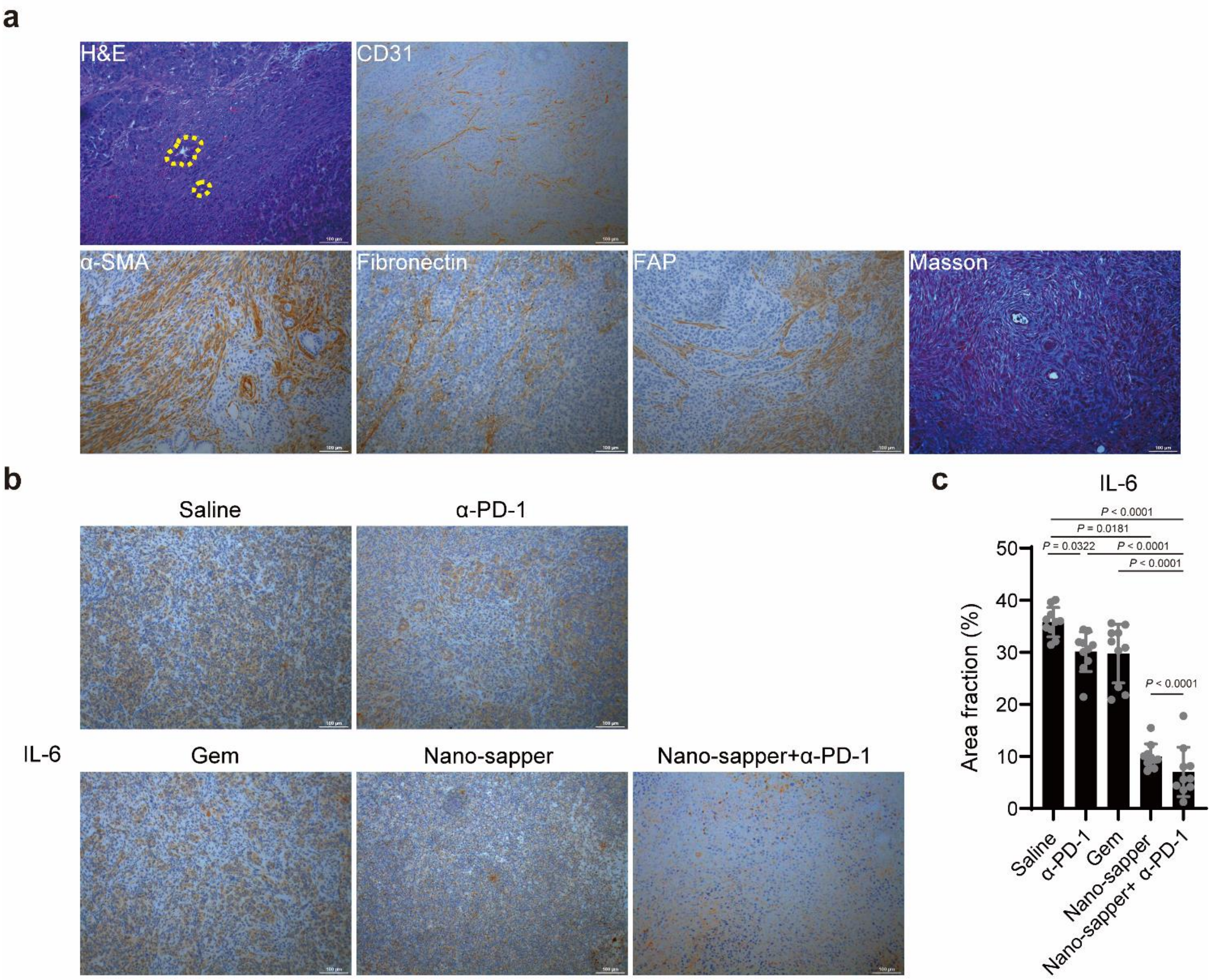

Supplementary Figure 20 Histopathological features of KPC1199 model and IL-6 in TME was downregulated by Nano-sapper. **a** Histopathological characterization of KPC1199 model. Scale bars, 100 μm. Yellow dash indicated the duct structure. **b,c** Nano-sapper reduced the expression of IL-6 ($n = 10$ section images from three mice). Data were pooled from two independent experiments. Scale bars, 100 μm. Data are presented as mean ± s.d. One-way ANOVA with Bonferroni multiple comparisons post-test was used for **c**. Error bars represent s.d..

Supplementary Table 1. Size and zeta potentials of nanoparticles.

Formulations	Size (nm)	Zeta potential (mV)	PDI
FHK-CaMP	36 ± 1.5	16 ± 0.2	0.173
FHK-pLIGHT@CaP	30 ± 0.4	13 ± 0.2	0.211
Nano-sapper	35 ± 2.1	15 ± 0.4	0.194
CaP	29 ± 1.7	13 ± 0.4	0.161

Data are presented as mean ± s.d.. (n = 3 independent experiments)

Supplementary Table 2. Median survival of orthotopic PDAC bearing mice (KPC1199).

Group	Median (day)	Log-rank test (VS)				
		Saline	Gem	α-PD-1	Nano-sapper	Nano-sapper+α-PD-1
Saline	27.5	\	\	\	\	\
Gem	34.5	0.0045	\	\	\	\
α-PD-1	29	0.4044	0.0190	\	\	\
Nano-sapper	41.5	0.0004	0.0055	0.0006	\	\
Nano-sapper+α-PD-1	52	0.0004	0.0005	0.0006	0.0022	\

Differences in survival were determined for each group by the Kaplan–Meier analysis with log-rank Mantel-Cox test (two-sided).

Supplementary Table 3. Median survival of orthotopic PDAC bearing mice (Panc02).

Group	Median (day)	Log-rank test (VS)				
		Saline	Gem	α-PD-1	Nano-sapper	Nano-sapper+α-PD-1
Saline	31	\	\	\	\	\
Gem	39	0.0066	\	\	\	\
α-PD-1	33.5	0.3024	0.0323	\	\	\
Nano-sapper	43	0.0005	0.0726	0.0006	\	\
Nano-sapper+α-PD-1	53.5	0.0005	0.0005	0.0006	0.0015	\

Differences in survival were determined for each group by the Kaplan–Meier analysis with log-rank Mantel-Cox test (two-sided).

Supplementary method. Antibodies used in this study.

Antibodies	Company	Catalog	Application and Dilution
Anti-CD31	Abcam	ab28364	IHC-F, 1/50
Anti-VE-cadherin	Abcam	ab33168	IHC-F, 1/100
Anti-ICAM-1	Abcam	ab171123	IHC-F, 1/100
Anti-MECA79	Biolegend	120801	IHC-F, 1/50
AF488 Goat Anti-Rabbit IgG H&L	Abcam	ab150077	IHC-F, 1/600
Anti-α-SMA	Abcam	ab5694	WB, 1/1000; IHC-P, 1/200
Anti-FAP	Abcam	ab207178	WB, 1/1000; IHC-P, 1/250
Anti-fibronectin	Abcam	ab23750	WB,1/1000 , IHC-P, 1/500
Anti-Smad2	Abcam	ab33875	WB, 1/2000
Anti-p-Smad2	Abcam	ab53100	WB, 1/1000
Anti-Smad3	Abcam	ab28379	WB, 1/1000
Anti-p-Smad3	Abcam	ab52903	WB, 1/2000
Anti-GAPDH	Abcam	ab8245	WB, 1/10000
Goat anti-rabbit HRP	Abcam	ab205718	WB, 1/25000
PE anti-mouse CD45	Biolegend	103106	FC, 1/100
APC anti-mouse CD45	Biolegend	103112	FC, 1/100
PerCP-Cy5.5-anti mouse CD3e	eBioscience	45-0031-82	FC, 1/20
AF647 anti-mouse CD8a	Biolegend	100724	FC, 1/200; IHC-F, 1/100
AF647 anti-mouse CD4	Biolegend	100424	IHC-F, 1/100
AF488 anti-mouse CD4	Biolegend	100423	FC, 1/1000; IHC-F, 1/100
Alexa Fluor 647 anti-mouse FoxP3	Biolegend	126408	FC, 1/50
AF594 anti-mouse B220	Biolegend	103254	FC, 1/200, IHC-F, 1/10
APC anti-mouse F4/80	Biolegend	123116	FC, 1/100
